# Charity campaigns with promotion-framed goals are more effective than those with prevention-framed goals

**DOI:** 10.1371/journal.pone.0286028

**Published:** 2023-08-02

**Authors:** Katarzyna Sekścińska, Agata Trzcińska, Dominika Maison

**Affiliations:** Faculty of Psychology, University of Warsaw, Warsaw, Poland; University of Gottingen, GERMANY

## Abstract

Proper communication with the public is crucial for encouraging private donors to make financial and non-financial donations to charities. This study compared the effectiveness of an advertising campaign that used a prevention framing for the charity’s purpose and one that used a promotional framing. This experimental study was conducted online with 547 participants. The results showed that the advertising message highlighting the promotional goals of the campaign was more effective than the one based on prevention goals. This result was observed not only for the evaluation of the campaign and organization as well as behavioral intentions, but, crucially, also on the level of actual behavior.

## 1. Introduction

Charities play an important role in addressing major social problems (such as poverty, hunger, health care, and inclusive and equitable education). However, to achieve their goals, organizations need money. One source of financial support is private donors, who account for over 70% of annual charitable giving in the United States [1]. Charities promote positive and desirable goals, but they can communicate these goals in different ways. In this article, we employ Regulatory Focus Theory [2, 3] to show that not only the goal of a charity campaign, but also the framing of the goal, is important for garnering support. Framing the message in terms of attaining a positive outcome (e.g., “providing access to drinking water”) is an example of a promotion-framed charity goal, while framing it as avoiding a negative outcome (e.g., “preventing drinking water shortages”) is a prevention-framed charity goal [2, 3]. In the present study, we analyze the effectiveness of promotion- and prevention-framed advertising goals in influencing people’s perceptions of a charity and a campaign, as well as their donation and volunteering intentions and behaviors.

In an experimental study, subjects were presented with one of two leaflets asking them to support a charity. One leaflet referred to a promotional charity goal, the other to a preventive goal. This study has added to the body of knowledge about how the goals of charity campaigns, preventive or promotional, affect their effectiveness. Unlike previous research, our study simultaneously analyzed a wide range of measures of charity campaign effectiveness. We were able to demonstrate that the advertising message that emphasized the promotional goals of the campaign was more effective than the one based on prevention goals. This result was observed for both campaign and organization evaluations and behavioral intentions to donate money and time. Importantly, we were able to demonstrate that framing of charitable messages also influences actual charitable behavior (investment of time and money).

## 2. Literature review

Regulatory Focus Theory put forward by Higgins [2, 3] states that people pursue their goals with different regulatory orientations, adopting either a promotion-framed or a prevention-framed orientation. The strategies for achieving goals differ in that the promotion focus is associated with striving for positive outcomes, whereas the prevention focus is concerned with avoiding negative outcomes [4]. Previous research has shown that an individual’s regulatory focus is related to their charitable behavior. Bullard and Penner [5] and Park and Ryu [6] showed that participants’ promotion focus positively predicted donation frequency, size of contributions, and the past year’s total donations to charity, while Choi and Park [7] found that stronger prevention focus was related to lower donation intention, the amount pre-committed to donate to charity, and the amount actually donated to charity.

However, it should be noted that people’s regulatory focus is formed not only by dispositional tendency (chronic regulatory focus) but also by situational factors, such as message framing [8–10]. The pioneers of message framing research are Tversky and Kahneman [11]. The studies on message framing showed that people react differently depending on whether they are confronted with gain- or loss-framed messages [e.g., 12, 13]. However, a meta-analysis conducted by Xu and Huang [14] revealed that findings on the effectiveness of gain-framed vs loss-framed appeals are inconsistent. This inconsistency of results can be explained by extending Kahneman and Tversky’s Prospect Theory [11] to include the Regulatory Focus Theory (RFT) posited by Higgins [8], which assumes that desirable goals (gain-framed goals) can be framed in terms of promotion or prevention–adding another dimension of framing. Thus, according to RFT, both gain- and loss-framed messages can also be promotional or preventive. Gain-framed messages may focus on achieving a positive outcome (promotion frame) or avoiding a negative outcome (prevention frame); for example, “with your help, more children will be healthy” (gain–promotion frame) versus “with your help, fewer children will suffer from illnesses” (gain–prevention frame). In contrast, loss-framed messages may focus on achieving a negative effect or failing to achieve a positive outcome [8]; for example, “without your help, more children will suffer from illnesses” (loss–promotion frame) vs. “without your help, fewer children will be healthy” (loss–prevention frame). In this study, we focus on gain-framed charity advertisements with promotion- and prevention-oriented messages.

Previous studies indicate that promotion-oriented persuasion campaigns are generally more successful than prevention-oriented campaigns [15]. Some studies have investigated this in the context of charity advertisements [16–18] and showed that promotion-framed charity appeals are perceived as more convincing and increase donation intention more than do prevention-framed charity appeals. Interesting results are also provided by Botner, Mishra, and Mishra [19], who showed that charities with a supportive orientation (promoting a cause; e.g., “Citizens for Urban Renewal”) are more likely to survive longer and receive more donations than charities that adopt a combative orientation (fighting against something; e.g., “Citizens Fighting Urban Decay”). Although the majority of studies show that promotion-framed charity appeals are more effective, it should be noted that one study found the opposite effect: Bullard and Penner [5] showed that while promotion focus motivates charitable giving among individuals, prevention-framed charity appeals are associated with greater declared willingness to support a cause than are promotion-framed appeals.

In general, the above studies suggest that promotion-oriented charity campaigns are more successful than prevention-oriented campaigns, when success is defined as a greater intention to support the campaign. However, taking into account the findings of the Money for Good study [20], which showed that one of the main barriers to increasing donation is potential donors’ skepticism and distrust of social sector actors, it seems important to measure the effectiveness of appeals for donations not only in terms of donation intentions, but also to analyze recipients’ perceptions of both charity campaigns and organizations. The results of previous research [5, 21] suggest that the more people feel the campaign may have an impact, the more willing they are to support a charity goal. In contrast, if they perceive the campaign as unnecessary and as *giving up resources*, they are less likely to help.

## 3. Hypotheses

This study aims to analyze the impact of different charity goal frames (promotion vs. prevention) on campaign perceptions. Based on the previously described research, we hypothesized that:

*H1*. *People evaluate charity campaigns with promotion-framed goals as being better than those with prevention-framed one*s.

At the same time, it is important for designers of charity advertisements to understand that many different aspects of charity campaigns can significantly affect the perception of their organization. Some previous research has shown that the perception of charity organizations may depend to some extent on how their advertising is presented. For example, Das, Kerkhof, and Kuiper [13] showed that the Dutch Leprosy Foundation was rated as being more relevant when its charity appeal was framed positively than when framed negatively. Based on the above-described research, we hypothesized that:

*H2*. *People perceive charities involved in campaigns with promotion-framed goals as being better than charities involved in campaigns with prevention-framed ones*.

Although some studies have indicated that donation intentions are powerful predictors of actual donations [22–24] and that intention to volunteer predicts subsequent reported volunteer behavior [25], many studies show that there exist discrepancies between declared and actual behavior and that measured intention does not always translate into behavior [26–28]. Given the above, it seems reasonable to ask whether a promotion-framed campaign will also be more effective than a prevention-framed campaign at influencing actual behavior. For this reason, our study introduced the measurement of not only behavioral intention but also actual behavior. We formulated two hypotheses related to the behavior dimension of charity campaign effectiveness:

*H3*. *People declare greater financial support (behavioral intention) to charity campaigns with promotion-framed goals than those with prevention-framed goals*.
*H4*. *People give more financial support (actual behavior) to charity campaigns with promotion-framed goals than those with prevention-framed ones*.

It is worth noting that charity manifests itself primarily in two areas of behavior: financial donations and volunteering (time donations). Most studies suggest that money and time donations are heterogeneous goods [29–31], as it is easier for some people to donate money and for others to donate time. Thus, specific charity advertisements may have a different impact on donation behavior than on volunteering behavior. Therefore, when analyzing the effectiveness of charity advertisements, it seems important to analyze both these aspects of charity behavior. The present study will analyze the effectiveness of different charity advertisements on various types of charity engagement. We formulated two hypotheses related to the behavior dimension of charity campaign effectiveness in the context of non-financial support:

*H5*. *People declare greater non-financial support (behavioral intention) to charity campaigns with promotion-framed goals than those with prevention-framed goals*.
*H6*. *People give more non-financial support (actual behavior) to charity campaigns with promotion-framed goals than those with prevention-framed ones*.

Additionally, measurement of both behavioral intentions and real behaviors allowed us to investigate how charity goal framing (promotion vs prevention) affects the actual implementation of charitable intentions, which was the next innovative element of this study. Promotion goals represent “the ideal self” (which includes hopes and aspirations) and therefore heighten one’s sensitivity to opportunities to advance goal attainment, while prevention goals represent the “ought self” (which includes duties and responsibilities) and heightens one’s sensitivity to impediments to goal attainment [32]. The actualization of a charitable intention seems to be more likely when the goal is promotion-framed (which focuses attention on goal attainment) than when the goal is prevention-framed–the increased sensitivity to impediments to goal attainment probably reduces the translation of charitable intentions into actual behaviors.

*H7*. *The discrepancy between the declaration to support a charity and actual behavior (both financial and non-financial) is smaller for campaigns with promotion-framed goals than for those with prevention-framed goals*.

In summary, this study aims to add to the body of scientific evidence on how prevention vs. promotion framed goals in charitable campaigns affect their effectiveness expressed in the assessment of the charity and the campaign and support for the campaign.

In order to take a broad look at the issue of supporting charities, we decided to analyze both financial and non-financial support. In addition, due to possible discrepancies between declared and actual support for the charity, we included both these measures of campaign effectiveness in the study.

## 4. Method

### 4.1. Participants

The participants were 547 Polish working adults (268 women and 279 men, aged 18–84 years; *M* = 44.84, *SD* = 16.14). Sensitivity analysis (G*Power) [33] revealed that the sample provided 80% power for detecting a small effect of Cohen’s *d* = 0.21 in a *t*-test for independent samples analyses.

The study was conducted using ARIADNA–an online Polish research panel with more than 150,000 registered participants. To test for response bias, there were two control questions on the survey in which the participants were asked to indicate particular numbers on a 7-point scale. Moreover, the time spent on the survey was controlled, with the lower limit of acceptable time established based on the average number of Polish words read in a minute by a Polish adult and the average time taken to answer a closed-ended question. The average drop-out level on the ARIADNA panel is 20%.

Informed written consent was obtained from all participants. As compensation for participating, participants were awarded points that were redeemable for various rewards offered by ARIADNA. The Ethics Board of the Faculty of Psychology at the University of Warsaw approved the study. The finer details of the methods employed and instructions given to participants are available from the authors upon request. No data were collected for any studies after the initial data analysis. No participants were excluded from any of the analyses.

### 4.2. Materials and procedure

Independent variable:

**Campaign goal frame** (promotion vs prevention)–experimental manipulation. In order to manipulate the frame of the campaign goal, two versions of a charity leaflet were prepared (see Supplementary Materials). Both leaflets publicize the (fictitious) Poland Help Foundation, which supports education-related causes. The leaflets differed in the description of the charity’s goals, presenting them using either promotion or prevention framings.

The text on the leaflets translated into English was as follows (bold fragments differed between versions of the leaflet–the version in the prevention frame is shown in parentheses; note that the English language allowed greater simplification of the sentences than in Polish, due to the broader meaning of words and different syntax):

“*Every fourth child in Poland* ***shows math talents [has difficulties with mathematics]*** *in the first year of school education*.

*Mathematical competences are essential in almost every sphere of life*.

*Supporting children **with mathematical talents [with math difficulties]** from the earliest stages of their education is important **for developing their talent [to avoid a build-up of difficulties]***.

*The funds collected by the charity will allow children **to achieve high results at school [meet the requirements for mathematical knowledge at school]** and, **by developing mathematical competences, will help them achieve positive outcomes in adult life [will protect them against negative outcomes in adult life].***

*Together, we can **develop the mathematical talents of children in Poland [prevent mathematical exclusion of children in Poland]”***

The charity presented in the ads (“Poland Help”) did not actually exist in order to avoid the charity’s image influencing the results. However, it was presented to respondents as an existing charity to increase the realism of the survey. Using such deception was necessary because it has been shown that a nonprofit’s brand image influences intentions to give time and money as well as actual giving behaviors [34, 35]. In particular, brand image explains up to 31% of intentions to give money and 24% of intentions to give time [35]. Thus, we assumed that using an existing charity in our study may bias the study results, leading to conclusions not fully resulting from the experimental manipulation, but at least partly from the image of charity or conscious and unconscious attitudes towards it. Information that the charity used in the study was fictitious was provided to the participants at the end of the study.

Dependent variables:

**Evaluation of the campaign–**participants were presented with 7 statements and asked to indicate the extent to which they agree with them on a scale from 1 (*definitely no*) to 7 (*definitely yes*). The first four statements measured the perceived *Impact* of possible support for the cause (e.g., “Getting involved in this campaign can do a lot of good for Polish children”). The last three statements measured the extent to which the participants perceived supporting the campaign as *Giving Up Resources* (e.g., “Getting involved in this action is ‘throwing money down the drain’”). The indicators for the Impact and Giving Up Resources variables were calculated as the mean of the answers for the relevant questions.

**Perception of the charity–**participants answered the following questions: “Do you think the Poland Help Foundation is (a) honest, (b) trustworthy, (c) professional, (d) profit-oriented, (e) dishonest, (f) unreliable.” They marked their answers on a scale from 1 (*definitely no*) to 7 (*definitely yes*). The indicator was calculated as the mean of the answers, with the answers for the last three adjectives reverse coded.

**Financial support for the charity** was measured on three levels: declared willingness to donate to the campaign, actual donation behavior related to the campaign, and declared willingness to donate to other campaigns from this charity.

- **Declared willingness to donate to the campaign**–participants were asked to imagine that they had just received PLN 100 (~22 USD) and were to divide this amount between private consumption and a donation to the charity on the leaflet.
- **Donation to the campaign (behavior)–**as part of the standard procedure of the ARIADNA panel, participants earned 30 points for participating in the study, which they could later exchange for several hundred products offered by the platform running the study; thus, the points can be treated as a money equivalent. In the experiment, respondents were asked if they would like to donate some or all of their points to the Poland Help campaign that was presented in the leaflet (*declaration to support the charity*), and if they answered “yes,” participants decided how many points to transfer to the Poland Help account (scale from 0 to 30 points–*actual behavior*).
  Based on the answers to these two questions, an additional indicator was built to measure the **discrepancy between declared financial support to the charity and actual behavior**, calculated as the difference between the declaration (1 –*ready to help*; 0 –*not ready to help*) and actual behavior (1 –*transferred at least 1 point to the charity;* 0 –*no transfer*).
- **Declared willingness to donate to other campaigns–**participants were asked to answer the question: “How likely do you think it is that you will become financially involved in campaigns other than those described in the Poland Help leaflet?”. Answers were given on a scale from 1 (*impossible*) to 7 (*certain*).

**Non-financial support for the charity** was measured on three levels: declared willingness to volunteer for the campaign, actual supportive behavior related to the campaign (volunteering), and declared willingness to volunteer for other campaigns.

- **Declared willingness to volunteer for the campaign–**participants were informed that the Poland Help Foundation needs volunteers to carry out the action presented in the leaflet. They were informed that volunteers carry out various types of tasks tailored to their skills and abilities. Participants were asked how much time they would be willing to spend to support the Poland Help campaign (answer given in full hours).
- **Volunteering (behavior)–**participants were asked to voluntarily (without extra panel-points) contribute to the Poland Help Foundation by creating some promotional slogans for the campaign. Firstly, they decided whether they would like to help or not (*declaration to support the charity*), and if they answered “yes,” they were presented with a text box in which they were to write the slogans. The time spent creating slogans was measured as an indicator of volunteering (behavior).
  On this basis, an indicator of *discrepancy between declared non-financial support for the charity and actual behavior* was built. The indicator was calculated as the difference between the number of participants who declared they would support the charity (answer: “yes, I would like to create the slogans now.”) and those who actually worked on the slogans (tried to write at least one slogan).
- **Declared willingness to volunteer for other campaigns–**participants were asked to answer the question: “How likely do you think it is that you would undertake voluntary activities in campaigns other than the one described in the Poland Help leaflet?”. The answers were given on a scale from 1 (*impossible*) to 7 (*certain*).

**Rating of the leaflet**–in order to control for possible difference (evaluative and emotional) between the two versions of the leaflet used in the experiment, participants were asked to rate the leaflet on seven dimensions presented as a 7-point semantic differential (e.g., *not interesting*–*interesting*, *unconvincing*–*convincing*). Based on the answers (means), a global indicator of general rating of the leaflet was created.

**Emotions evoked by the leaflet–**Participants were asked to assess the feelings the Poland Help leaflet evoked in them. They rated six basic emotions (based on Ekman, [36]) on a 7-point scale (1 –*I definitely did not feel this* to 7 –*I definitely felt this*).

### 4.3. Procedure

After completing the sociodemographic data, each participant was randomly assigned to one of the experimental conditions (promotion frame, *n* = 262; prevention frame, *n* = 285). Randomization did not assign exactly the same number of participants to each group, but the difference in size between the groups of participants was not statistically significant–*χ* ^*2*^(1) = 0.97, *p* = .33. Participants were then presented with one version of the Poland Help campaign leaflet and completed the declarative behavioral intention measures, perception of the charity measures, rating of the leaflet measures, evaluation of campaign measures, and finally the donation behavior and voluntary work measures. At the end of the procedure, all participants were fully debriefed. They were informed that the charity described in the study did not actually exist and were told why a real charity was not used. In addition, the participants were informed that the points they donated to the fictitious charity remained in their Ariadna on-line panel accounts (so they didn’t lose anything) and that the remuneration for participating in the study was estimated by the organizers taking into account the time needed to complete the last task related to advertising slogans.

## 5. Results

A series of *t*-tests and McNemar’s test were conducted to analyze the differences between the two experimental groups in terms of perception of the campaign, perception of the charity, declared propensity to support the charity, actual charity behavior, and rating of the leaflet.

To control the false discovery rate for multiple hypothesis testing, the Benjamini–Hochberg (B-H) method was used [37]. The critical B-H for each variable is given in the Supplementary Materials. Note that all the significant differences between the experimental groups that occurred in the present analyses remained significant after taking into account the critical B-H.

The additional regression models for each dependent variable with age and sex introduced into the models has also been conducted. The results are presented in the Supplementary Materials.

### 5.1. Manipulation check

To be sure that the possible differences in reactions to the leaflet between the promotion-framed and prevention-framed groups were not due to different perceptions of the leaflet or different emotional reactions to the leaflet, a series of *t*-test analyses were conducted. The results showed no significant differences between groups in their ratings of the leaflet on all seven analyzed dimensions and no significant differences in levels of the six analyzed emotions potentially evoked by the leaflet. The data analyses are presented in the Supplementary Materials.

### 5.2. Evaluation of the campaign

Two *t*-test analyses were conducted to verify whether people evaluate charity campaigns with promotion-framed goals as being better than those with prevention-framed ones (H1). There was a significant difference observed between groups in terms of their evaluation of the campaign as an action that can make an Impact–*t*(545) = 2.473, *p* = .014. Participants from the promotion-framed experimental group (*M* = 4.57, *SD* = 1.43) scored higher on this indicator than those from the prevention-framed group (*M* = 4.25, *SD* = 1.57). However, no significant difference between the groups appeared in terms of the evaluation of the campaign as an action related to Giving Up Resources–*t*(545) = 0.640, *p* = .522; *M*_promotion_ = 3.59, *SD*_promotion_ = 1.45; *M*_prevention_ = 3.51, *SD*_prevention_ = 1.52.

### 5.3. Perception of the charity

A series of *t*-test analyses were conducted to verify whether people perceive charities involved in campaigns with promotion-framed goals as being better than charities involved in campaigns with prevention-framed ones (H2). It was found that participants from the promotion-framed group generally rated the charity higher than participants from the prevention-framed group (Table 1). Further analyses showed that the promotion-framed group perceived the charity as being better on all positive dimensions (honest, trustworthy, and professional), but no differences were observed in the context of the negative dimensions (profit-oriented, dishonest, and unreliable).

**Table 1:** Differences between experimental groups in terms of perception of the charity.

	Total	Promotion frame	Prevention frame			
	*M (SD)*	*M (SD)*	*M (SD)*	*t*	*df*	*p*
General indicator	4.40 (1.51)	4.57 (1.43)	4.25 (1.57)	2.442	545	.015
Honest	4.27 (1.48)	4.47 (1.36)	4.09 (1.55)	3.044	545	.002
Trustworthy	4.33 (1.45)	4.50 (1.35)	4.17 (1.52)	2.690	545	.007
Professional	4.39 (1.49)	4.53 (1.42)	4.26 (1.54)	2.137	545	.033
Profit-oriented	3.85 (1.56)	3.75 (1.49)	3.94 (1.61)	-1.419	545	.156
Dishonest	3.17 (1.48)	3.09 (1.43)	3.24 (1.53)	-1.189	545	.235
Unreliable	3.33 (1.55)	3.27 (1.49)	3.37 (1.60)	-0.732	545	.464

Note: the results of t-test analyses are presented

### 5.4. Financial support for the charity

Further analyses investigated whether people declare greater financial support (behavioral intention; H3) and if they give more financial support (actual behavior; H4) to charity campaigns with promotion-framed goals than those with prevention-framed goals.

The results showed that, on both the declarative and behavioral levels, the differences between experimental groups in their financial support of the campaign were significant (Table 2). Participants who saw the promotion-framed leaflet declared greater willingness to donate to the campaign and transferred more panel points to the charity account than did those who saw the prevention-framed leaflet. However, there were no significant differences observed in the number of people from both groups who answered “yes” to whether they would be willing to transfer part of their points to the charity account–*χ* ^*2*^(1) = 0.103, *p* = .749.

**Table 2:** Differences between experimental groups in terms of declared and actual financial support for the charity.

	Total	Promotion frame	Prevention frame			
	*M (SD)*	*M (SD)*	*M (SD)*	*t*	*df*	*p*
Declared willingness to donate the campaign (in PLN)	31.47 (27.93)	34.79 (27.92)	28.42 (27.64)	2.681	545	.008
Donation to the campaign (behavior; panel points)	19.06 (8.42)^a^	20.85 (7.00)^a^	17.34 (9.31)^a^	2.613	141	.010
Declared willingness to donate other campaigns	3.43 (1.60)	3.61 (1.59)	3.26 (1.60)	2.577	545	.010

Note: the results of t-test analyses are presented, ^a^ only participants who decided to donate to the charity

Then, analyses were conducted to test whether the discrepancy between the declaration to financially support a charity and actual behavior is smaller for campaigns with promotion-framed goals than for those with prevention-framed goals (H7). The hypothesis was not confirmed by the results. It was found that participants who declared willingness to transfer some part of their remuneration for participating in the study to the charity account actually did so. No significant drop-out was observed for the promotion-framed group– 0% drop-out, 74 participants declared they would help, 188 refused, and all 74 actually helped; McNemar’s *χ* ^*2*^(1) = 0, *p* = 1.000. Nor was a significant drop-out observed for the prevention-framed group– 0.004% drop-out, 77 participants declared they would help, 208 refused, and 76 people actually helped; McNemar’s *χ* ^*2*^(1) < 0.001, *p* = 1.000.

However, additional analysis of declared willingness to engage in other Poland Help Foundation campaigns showed that participants who were exposed to the promotion-framed leaflet assessed the probability of their donating to other campaigns higher than did the other group (Table 2).

### 5.5. Non-financial support for the charity

Further analyses aimed to verify whether people declare greater non-financial support (behavioral intention, H5) and if they give more non-financial support (actual behavior, H6) to charity campaigns with promotion-framed goals than those with prevention-framed goals.

There were significant differences between the groups in terms of the time that participants declared they would be willing to spend volunteering for the charity and the actual time spent doing so (Table 3). The promotion-framed group declared being willing to spend more time on voluntary work and actually spent more time creating campaign slogans than did the prevention-framed group.

**Table 3:** Differences between experimental groups in terms of declared and actual volunteering support for the charity.

	Total	Promotion frame	Prevention frame			
	*M (SD)*	*M (SD)*	*M (SD)*	*t*	*df*	*p*
Declared willingness to volunteer for the campaign (in hours)	5.09 (15.87)	6.79 (21.13)	3.53 (8.30)	2.337	334	.020
Volunteering (behavior; in seconds)	133.37 (103.07)^a^	149.50 (103.32)^a^	117.23 (102.82)^a^	2.185	194	.030
Declared willingness to volunteer for other campaigns	3.15 (1.68)	3.35 (1.67)	2.96 (1.67)	2.758	545	.006

Note: the results of t-test analyses are presented; ^a^ only participants who decided to volunteer for the campaign; The very large SD for declared willingness to volunteer was due to four extremely high answers– 2 participants declared 100 hours and 2 declared 200 hours.

No significant differences were observed in the number of people from both groups who answered “yes” to the question of whether they would like to help with slogans–*χ* ^*2*^(1) = 2.776, *p* = .096. However, not all participants who declared their willingness to help writing slogans actually did so. Further analyses aimed to verify whether the discrepancy between the declaration to non-financially support a charity and actual behavior was smaller for campaigns with promotion-framed goals than for those with prevention-framed goals (H7). There was a significant drop-out observed in the prevention-framed group– 12% drop-out, 121 participants declared they would help, 164 refused, but only 106 people actually helped, while 179 did not; McNemar’s *χ* ^*2*^(1) = 13.067, *p* = .0003. There was a non-significant drop-out in the promotion-framed group– 3% drop-out, 93 participants declared to help, 169 refused, and 90 people actually helped, while 172 did not; McNemar’s *χ* ^*2*^(1) = 1.333, *p* = .248. Moreover, further analyses showed a significant difference in drop-out for non-financial support between the two experimental groups–*χ* ^*2*^(1) = 7.12, *p* = .008.

Moreover, the additional analysis of declared willingness to volunteer for other Poland Help Foundation campaigns showed that participants who were exposed to the promotion-framed leaflet declared greater willingness to engage than did the other group (Table 3).

### 5.6. The relationship between financial and non-financial support for the charity

Finally, we conducted additional analyses of correlations between variables related to financial and non-financial support for the charity, to verify that money and time donations are heterogenous goods (Table 4). Declared willingness to donate to the campaign (in PLN) correlated positively, albeit very weakly, with actual donation to the campaign and with declared willingness to volunteer for the campaign. No other significant correlations were observed.

**Table 4:** Pearson correlations between variables pertaining to financial and non-financial support for the charity.

	*2*	*3*	*4*
1. Declared willingness to donate the campaign (in PLN)	.18*	.12**	.03
2. Donation to the campaign (behavior; panel points)		.08	-.03
3. Declared willingness to volunteer for the campaign (in hours)			-.07
4. Volunteering (behavior; in seconds)			

Note: the results of Pearson correlation analyses are presented; * *p* < .05;** *p* < .01; the columns refer to the categories in the rows.

## 6. Discussion

Previous studies, although sparse [16, 17], analyzed how promotion- vs prevention-framing of charity goals affects the effectiveness of social campaigns, operationalized as donation intention. Our results expand the literature by showing that promotion-framing of charity goals (compared to prevention-framing) leads to: (1) higher evaluation of the campaign as an action that can make an impact and (2) better perception of the charity (more positive; i.e., honest, trustworthy, and professional). Thus, it seems that decisions about what activities a charity engages in and how it talks about them has consequences–not only for the effectiveness of the campaign itself, but also for the perception of the charity that organizes the campaign.

A very important and novel finding of our study, distinguishing it from previous work, is that our measurement of the effectiveness of the advertising (the dependent variable) was operationalized not only in terms of behavioral intention, but also actual behavior. The respondents had the option to allocate part or all of their remuneration for participating in the study to the campaign presented in the study (financial donation to the campaign). The obtained results showed that compared to the prevention-framing, promotion-framing of the campaign goal increased financial support for the charity, not only on the declarative level (declared willingness to donate to the campaign) but also on the level of actual behaviors (actually donating more to the campaign).

Moreover, the obtained results showed that the greater effectiveness of promotion-framed goals than prevention ones may be observed not only in the context of charitable donation (financial support) but also in context of non-financial support (i.e., giving other resources like time, knowledge, abilities, etc.), which was operationalized in this study as volunteering. The results of the present study indicate that, similarly to the case of financial support, both declared non-financial support (declared willingness to volunteer for the campaign and other campaigns by the same charity) and actual non-financial support (real behavior; i.e., time spent volunteering) were greater for the promotion-framed goal than the prevention-framed one.

Taken together, our results replicate some previous studies showing that promotion-framed goals (compared to prevention-framed ones) lead to greater financial charity donation intention [16, 17], but expand them by (1) showing that goals framed thusly also lead to larger declared non-financial support (voluntary work) and (2) are also more effective when it comes to actual charity behaviors (both financial and non-financial). Yet another interesting result of the present study is that the discrepancy between declaration of intention to support the charity and actual behavior was smaller for the campaign with the promotion-framed goal than the one with the prevention-framed goal; however, this was true only in the case of non-financial support for the charity.

The results showed also that when the charity goal was promotion-framed, almost everyone who declared being willing to volunteer actually helped by engaging in writing slogans (a nonsignificant 3% drop-out was observed), whereas a significant drop-out (12%) was observed when the goal was prevention-framed. This means that when the goal was preventive, a significant number of people who said they were willing to volunteer did not translate their declaration into actual behavior. Importantly, there was no difference between experimental conditions (promotion- vs prevention-framed goals) in terms of the number of people who declared being willing to help create slogans (behavioral intention to volunteer), but there was a difference in actual involvement in creating the slogan (actual behavior). This result is of great importance in the context of advertising pre-testing studies, which, as previously mentioned, are often based on measurements of declared behavior [16–18]. Our research has shown that these results are sometimes overstated when compared to real behavior.

Nevertheless, it should be noted that the majority of people who declared being willing to help the charity (financially or non-financially) actually helped, but in the case of non-financial support, the discrepancy between declaring being willing to support the charity and actual behavior was greater than in the case of financial support. Although more people declared being willing to volunteer than to give financial support, the dropout in the case of financial support was smaller and almost non-existent. This was probably because the task of actually donating points to the charity (financial support) was much easier and less time-consuming than devoting time to creating slogans supporting the charity campaign. Thus, it appears that an analysis of charity campaign effectiveness based only on participant intentions may be inadequate, especially when the charity action (e.g., volunteering) requires a greater commitment (e.g., time).

The results of this study have many practical implications. Unfortunately, in recent years, significant drops in charitable giving and volunteering have occurred in many developed countries (even those with well-established charitable traditions and highly developed civic infrastructure) [38]. Therefore, it is extremely important to understand how to formulate messages that effectively encourage people to donate money to an organization or otherwise support it (e.g., by volunteering). The result indicating that promotion-framed charity goals are more effective than prevention-framed goals is of great importance for practitioners (i.e., creators of charity advertising). Many advertising professionals believe that negative emotions are a more effective means of persuasion in social advertising than are positive emotions [39]. However, the use of negative emotions may lead to campaign goals being formulated in a preventive manner (focused towards avoiding negative outcomes), which, according to our results, may reduce the campaign’s effectiveness.

In line with previous studies [27, 28], another practical implication of the present research is that charity advertising pre-testing should be based not only on measurement of behavioral intentions, but also on real behaviors (as far as possible), because behavioral intentions may not always translate into behavior.

Furthermore, there are two other important practical implications of the present study. Firstly, the study confirms previous reports that money and time donations are heterogenous goods [29, 30], and showed that there is a very weak relationship between declaration to donate to a campaign and declaration to volunteer for a campaign and that there is no relationship between actual donation and volunteer behavior. Secondly, the study demonstrated that promotion and prevention framings have different effects for financial and non-financial support of charities. These results have significant practical implications, because, depending on the purpose of the campaign (financial vs. non-financial support), different messages or ways of formulating messages may be more or less effective.

Although the obtained results seem promising, several limitations of this study

should be acknowledged. The study used a fictitious charity that was presented as real. This made it possible to limit the impact of the charity’s image on the results of the study, but at the same time it caused a small (but notable) risk that the participants of the study might realize the charity to be fake. If that were the case, their answers might be biased. Moreover, when measuring the declared financial support to the campaign, we asked participants to imagine that they had just received PLN 100 that they could spend on charity, and therefore analyzed their hypothetical decisions. However, this instruction was necessary because it allowed us to control the size of the pool from which respondents chose the amount of financial support for the charity. Furthermore, the tools used in the study to measure the declared and actual time spent helping the charity might not be fully comparable. The task that the respondents were actually supposed to perform may have appeared more pleasant and easier than those that came to mind when answering the less specific question about declared willingness to volunteer for other campaigns.

A previous study by Maison [40] showed that it makes no difference whether a charity’s support is intended for gifted children or for children with learning difficulties if it is directed at people of the same nationality as the respondent (Polish, as in our study). The results of this study showed that help for gifted children and help for children with learning difficulties were rated equally on the following dimensions: (a) evaluation of the intervention, (b) perception of the importance of the problem, and (c) perception of the charity. Our experimental manipulation was developed based on the above research findings, but it can be considered a limitation that our manipulation changed not only the framing of the advertising message but also the target of the charity. In the condition with the promotion framed goal, gifted children were supported, whereas in the condition with the prevention framed goal children with learning problems were supported. However, based on Maison’s [40] findings, we can assume that Poles are equally willing to help gifted Polish children and those with learning difficulties. Nevertheless, in the future it will be worthwhile to conduct more detailed studies in which promotion and prevention framed goals are targeted to different groups of recipients (e.g., gifted children and children with learning difficulties).

This study also suggests new directions for further research. Firstly, it seems important to conduct broader analyses in which other important characteristics of charity goals (both promotion- and prevention-framed) are analyzed, including, inter alia: (1) referring to positive and negative emotions, as Zemack-Rugar and Klucarova-Travani [17] showed that promotion-framed messages raise donation intentions when combined with happy images but not with sad images; (2) short-term and long-term charity goals; (3) loss framed goals (e.g., “without your help fewer children will develop their mathematical talents”); and (4) goals addressing various social problems and concerning different social groups.

Another interesting path for further research is to investigate whether the observed results showing the greater effectiveness of promotion over prevention framings may be applicable to corporate social responsibility campaigns. In this context, it would be worth investigating whether different campaign goal framings (promotion vs prevention) affect the perception of a company’s products, the willingness of consumers to buy its products, and real consumer behaviors (real consumer choices).

In conclusion, the present well-powered experimental study conducted with working adults indicated that charity campaigns with promotional goals may be more effective than campaigns with preventive ones. Moreover, the greater effectiveness of such campaigns may manifest not only in terms of evaluations of the campaign and associated charity or behavioral intentions but, crucially, also on the level of actual behavior (investing time and money).

## Supporting information

S1 File Experimental manipulation materials, manipulation check analyses, false discovery rate and critical b-h analyses and additional regression models for each dependent variable with age and sex included in the models. (DOCX)
