# Supplementary material for: Charity campaigns with promotion-framed goals are more effective than those with prevention-framed goals
Source: PLoS One. 2023 Aug 2;18(8):e0286028. doi: 10.1371/journal.pone.0286028 (PMC10395818; doi:10.1371/journal.pone.0286028)
Supplement: S1 File — (DOCX) [file pone.0286028.s001.docx]

**Charity campaigns with promotion-framed goals are more effective than those with preventive-framed goals**

**SUPPORTING INFORMATION**

**EXPERIMENTAL MANIPULATION MATERIALS**

1. **Promotion-framed goal experimental condition**

**
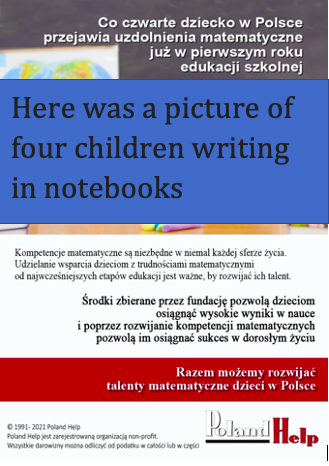
**

**Translation of the text on the leaflet:**

Every fourth child in Poland shows math talents in the first year of education

Mathematical competences are essential in almost every sphere of life.

Supporting children with mathematical talents from the earliest stages of their education is important for developing their talent.

The funds collected by the foundation will allow children to achieve high results at school and, by developing mathematical competences, will allow them to achieve success in adult life.

Together, we can develop the mathematical talents of children in Poland

1. **Prevention-framed goal experimental condition**

**
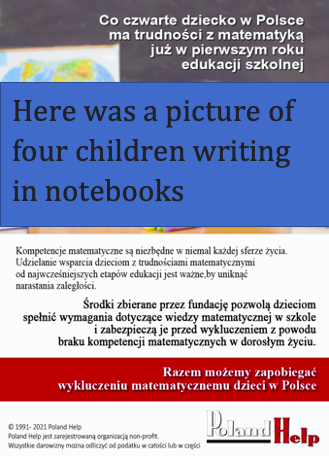
**

**Translation of the text on the leaflet:**

Every fourth child in Poland has difficulties with mathematics by the first year of school education

Mathematical competences are essential in almost every sphere of life.

Providing support to children with math difficulties from the earliest stages of education is important to avoid a build-up of difficulties.

The funds collected by the foundation will allow children to meet the requirements for mathematical knowledge at school and will protect them against exclusion due to lack of mathematical competences in adulthood.

Together, we can prevent mathematical exclusion of children in Poland

**MANIPULATION CHECK ANALYSES**

To be sure that possible differences observed in reactions to the leaflet presenting the campaign between the promotion-framed and prevention-framed groups were not due to different perceptions of the leaflet or different emotional reactions to the leaflet, a series of *t*-test analysis were conducted.

**Research tools:**

Rating of the leaflet - the participants were asked to rate the leaflet on 7 dimensions presented on semantic differential scales: 1 (*I don’t like it*) – 7 (*I like it*), 1 (*arouses negative emotions*) –7 (*arouses positive emotions*); 1 (*does not provide the necessary information*) – 7 (*provides the necessary information*); 1 (*uninteresting*) – 7 (*interesting*); 1 (*unconvincing*) – 7 (convincing); 1 (*does not encourage campaign support*) – 7 (*encourages campaign support*); 1 (*unprofessional*) – 7 (*professional*). The questions were analyzed one by one and the indicator (mean of the answers) of the general rating of the leaflet was calculated.

Emotions evoked by the leaflet **–** Participants were asked to assess the feelings the Poland Help leaflet evoked in them. They rated six basic emotions distinguished by Eckman (1972) on a scale from 1 (*I definitely did not feel it*) to 7 (*I definitely felt it*), namely: happiness, sadness, disgust, fear, surprise, and anger.

**Results:**

Rating of the leaflet

The results showed no significant differences between the groups in their ratings of the leaflet on all 7 analyzed dimensions (Table S1).

**Table S1. Descriptive statistics and *t*-test analyses for ratings of the leaflet**

|  | Promotion frame | Prevention frame | *t* |
| --- | --- | --- | --- |
| Leaflet rating - general indicator | 4.44 (1.36) | 4.25 (1.46) | *t*(545) = 1.584, *p* = .114 |
| (1) I don’t like it – (7) I like it | 4.43 (1.52) | 4.18 (1.59) | *t*(545) = 1.894, *p* = .059 |
| (1) arouses negative emotions – (7) arouses positive emotions | 4.37 (1.56) | 4.25 (1.61) | *t*(545) = 0.863, *p* = .389 |
| (1) does not provide the necessary information –  (7) provides the necessary information | 4.64 (1.52) | 4.44 (1.63) | *t*(545) = 1.471, *p* = .142 |
| (1) uninteresting –  (7) interesting | 4.51 (1.47) | 4.29 (1.62) | *t*(545) = 1.686, *p* = .092 |
| (1) unconvincing –  (7) convincing | 4.35 (1.56) | 4.12 (1.69) | *t*(545) = 1.612, *p* = .108 |
| (1) does not encourage campaign support –  (7) encourages campaign support | 4.29 (1.62) | 4.08 (1.75) | *t*(545) = 1.478, *p* = .140 |
| (1) unprofessional –  (7) professional | 4.48 (1.49) | 4.36 (1.58) | *t*(545) = 0.851, *p* = .395 |

Note: Columns 2 and 3 of the table present means with standard deviations in parentheses.

Emotions evoked by the leaflet

The results showed no significant differences between groups in the level of any of six analyzed emotions evoked by the leaflet (Table S2).

**Table S2.**

**Descriptive statistics and *t*-test analyses for emotions evoked by the leaflet**

|  | **Promotion frame** | **Prevention frame** | ***t*** |
| --- | --- | --- | --- |
| Happiness | 3.97 (1.56) | 3.77 (1.66) | *t*(545) = 1.486, *p* = .139 |
| Sadness | 2.98 (1.45) | 3.21 (1.47) | *t*(545) = -1.805, *p* = .072 |
| Disgust | 2.45 (1.48) | 2.54 (1.48) | *t*(545) = -0.727, *p* = .468 |
| Fear | 2.43 (1.45) | 2.48 (1.42) | *t*(545) = -0.403, *p* = .687 |
| Surprise | 3.63 (1.50) | 3.78 (1.62) | *t*(545) = -1.165, *p* = .244 |
| Anger | 2.83 (1.54) | 3.07 (1.57) | *t*(545) = -1.822, *p* = .069 |

Note: Columns 2 and 3 of the table present means with standard deviations in parentheses.

**FALSE DISCOVERY RATE AND CRITICAL B-H**

**Table S3.**

**Critical B-H for each Dependent Variable**

| **Variables** | ***p*** | **Rank** | ***Critical B-H*** |
| --- | --- | --- | --- |
| Perception of the foundation - Honest | .002 | 1 | .004 |
| Declared willingness to volunteer for other campaigns | .006 | 2 | .008 |
| Perception of the foundation -Trustworthy | .007 | 3 | .012 |
| Declared willingness to donate the campaign (*in PLN*) | .008 | 4 | .015 |
| Donation to the campaign (behavior; *panel points*) | .010 | 5 | .019 |
| Declared willingness to donate other campaigns | .010 | 6 | .023 |
| Perception of the foundation -General indicator | .015 | 7 | .027 |
| Declared willingness to volunteer for the campaign (*in hours*) | .020 | 8 | .031 |
| Volunteering (behavior; *in sec*) | .030 | 9 | .035 |
| Perception of the foundation -Professional | .033 | 10 | .038 |
| Perception of the foundation -Profit-oriented | .156 | 11 | .042 |
| Perception of the foundation -Dishonest | .235 | 12 | .046 |
| Perception of the foundation -Unreliable | .464 | 13 | .050 |

**Bibliography:**

[1] Benjamini, Y., & Hochberg, Y. Controlling the false discovery rate: a practical and powerful approach to multiple testing. *Journal of the Royal statistical society: series B (Methodological)*. 1995; 57(1), 289-300.

**ADDITIONAL REGRESSION MODELS FOR EACH DEPENDENT VARIABLE WITH AGE AND SEX INCLUDED IN THE MODELS**

**Evaluation of the campaign - Making impact**

|  | *B* | *SE* | *t* | *p* |
| --- | --- | --- | --- | --- |
| Campaign goal frame | -0.31 | 0.13 | -2.41 | .02 |
| Sex | -0.21 | 0.13 | -1.66 | .10 |
| Age | 0.001 | 0.004 | 0.31 | .76 |
| Constant | 5.15 | 0.34 | 15.19 | <.001 |
| *R^2^ = .02 ; F(3,543) = 3.032, p = .03* | | | | |

**Evaluation of the campaign – Giving up resources**

|  | *B* | *SE* | *t* | *p* |
| --- | --- | --- | --- | --- |
| Campaign goal frame | -0.08 | 0.13 | -0.63 | .53 |
| Sex | 0.13 | 0.13 | 1.04 | .30 |
| Age | -0.01 | 0.004 | -2.01 | .05 |
| Constant | 3.83 | 0.33 | 11.45 | <.001 |
| *R^2^ = .01; F(3,543) = 2.004, p = .11* | | | | |

**Perception of the charity**

|  | *B* | *SE* | *t* | *p* |
| --- | --- | --- | --- | --- |
| Campaign goal frame | 0.02 | 0.09 | 0.21 | .83 |
| Sex | -0.13 | 0.09 | -1.48 | .14 |
| Age | -0.001 | 0.003 | -0.27 | .79 |
| Constant | 4.65 | 0.24 | 19.53 | <.001 |
| *R^2^ = .004 ; F(3,543) = 0.738, p = .53* | | | | |

**Financial support for the charity**

1. **Declared willingness to donate to the campaign**

|  | *B* | *SE* | *t* | *p* |
| --- | --- | --- | --- | --- |
| Campaign goal frame | -6.48 | 2.35 | -2.76 | .006 |
| Sex | -3.37 | 2.36 | -1.43 | .15 |
| Age | 0.28 | 2.36 | -1.43 | <.001 |
| Constant | 33.88 | 6.16 | 5.50 | <.001 |
| *R^2^ = .05 ; F(3,543) = 8.453, p < .001* | | | | |

1. **Donation to the campaign (behavior)**

|  | *B* | *SE* | *t* | *p* |
| --- | --- | --- | --- | --- |
| Campaign goal frame | -3.86 | 1.36 | -2.84 | .01 |
| Sex | -0.15 | 1.36 | -0.11 | .91 |
| Age | 0.08 | 0.04 | 1.91 | .06 |
| Constant | 21.43 | 3.44 | 6.23 | <.001 |
| *R^2^ = .07 ; F(3,147) = 3.588, p = .02* | | | | |

1. **Declared willingness to donate to other campaigns**

|  | *B* | *SE* | *t* | *p* |
| --- | --- | --- | --- | --- |
| Campaign goal frame | -0.34 | 0.14 | -2.47 | .01 |
| Sex | -0.19 | 0.14 | -1.39 | .17 |
| Age | -0.01 | 0.004 | -1.90 | .06 |
| Constant | 4.59 | 0.36 | 12.84 | <.001 |
| *R^2^ = .02 ; F(3,543) = 3.915, p = .009* | | | | |

**Non-financial support for the charity**

1. **Declared willingness to volunteer for the campaign**

|  | *B* | *SE* | *t* | *p* |
| --- | --- | --- | --- | --- |
| Campaign goal frame | -3.181 | 1.36 | -2.35 | .02 |
| Sex | -1.59 | 1.36 | -1.17 | .24 |
| Age | -0.02 | 0.04 | -0.37 | .24 |
| Constant | 13.02 | 3.56 | 3.66 | <.001 |
| *R^2^ = .01 ; F(3,543) = 2.411, p = .07* | | | | |

1. **Volunteering (behavior)**

|  | *B* | *SE* | *t* | *p* |
| --- | --- | --- | --- | --- |
| Campaign goal frame | -39935.50 | 14235.92 | -2.81 | .01 |
| Sex | 2829.71 | 14137.04 | 0.20 | .84 |
| Age | 2081.33 | 453.47 | 4.59 | <.001 |
| Constant | 95699.65 | 35435.97 | 2.70 | .01 |
| *R^2^ = .12 ; F(3,192) = 8.773, p < .001* | | | | |

1. **Declared willingness to volunteer for other campaigns**

|  | *B* | *SE* | *t* | *p* |
| --- | --- | --- | --- | --- |
| Campaign goal frame | -0.37 | 0.14 | -2.64 | .01 |
| Sex | -0.17 | 0.14 | -1.16 | .25 |
| Age | -0.02 | 0.004 | -3.52 | <.001 |
| Constant | 4.66 | 0.37 | 12.55 | <.001 |
| *R^2^ = .04 ; F(3,543) = 6.932, p < .001* | | | | |
